# Engineered circular RNA-based DLL3-targeted CAR-T therapy for small cell lung cancer

**DOI:** 10.1186/s40164-025-00625-8

**Published:** 2025-03-12

**Authors:** Jingsheng Cai, Zheng Liu, Shaoyi Chen, Jingwei Zhang, Haoran Li, Xun Wang, Feng Yang, Shaodong Wang, Xiao Li, Yun Li, Kezhong Chen, Jun Wang, Ming Sun, Mantang Qiu

**Affiliations:** 1 Thoracic Oncology Institute, Peking University People’s Hospital, Beijing, 100044 China; 2 Department of Thoracic Surgery, Peking University People’s Hospital, No. 11 Xizhimen South Street, Xicheng District, Beijing, 100044 China; 3 Research Unit of Intelligence Diagnosis and Treatment in Early Non-small Cell Lung Cancer, Chinese Academy of Medical Sciences, 2021RU002, Peking University People’s Hospital, Beijing, 100044 China; 4 Institute of Advanced Clinical Medicine, Peking University, Beijing, 100191 China; 5 Department of Thoracic Surgery, Sichuan Clinical Research Center for Cancer, Sichuan Cancer Hospital& Institute, Sichuan Cancer Center, University of Electronic Science and Technology of China (UESTC), Chengdu, 610041 China; 6 Department of Oncology Center, The Affiliated Suzhou Hospital of Nanjing Medical University, Suzhou Municipal Hospital, No. 16 Baita West Road, Suzhou, 215001 China

**Keywords:** Circular RNA, Electroporation, CAR-T, Delta-like Ligand 3, Small cell lung cancer

## Abstract

**Purpose:**

Circular RNA (circRNA) has emerged as a promising RNA therapeutic molecule due to its enhanced stability and prolonged protein expression compared to messenger RNA (mRNA). Using circRNA to construct transient Chimeric Antigen Receptor (CAR)-T cells can mitigate the limitations of conventional viral vector-based CAR-T approaches, such as complex process and long-term side effects.

**Methods:**

The study first reconfirmed the advantageous properties of circRNA, focusing on its stability and protein expression efficiency. Electroporation conditions were then optimized for the efficient delivery of circRNA into human primary T cells. Subsequently, a circRNA encoding the anti-Delta-like Ligand 3 (DLL3) CAR was constructed, and CAR-T cells were generated via electroporation. The efficacy of circRNA-based CAR-T cells was compared to mRNA-based CAR-T cells in both in vitro and in vivo models, including subcutaneous and orthotopic small cell lung cancer (SCLC) mouse models.

**Results:**

CircRNA-based CAR-T cells demonstrated superior efficacy against SCLC compared to mRNA-based CAR-T cells. In vitro experiments showed enhanced tumor-killing effects, while in vivo studies revealed complete elimination of human SCLC tumors in both subcutaneous and orthotopic mouse models. These results underscored the therapeutic advantages of circRNA in CAR-T cell therapy.

**Conclusions:**

This study validated the feasibility of the circRNA-electroporation strategy in CAR-T cell therapy and offered a potentially effective approach for treating SCLC, highlighting the potential of circRNA-based technologies in advancing cell therapies.

**Graphic Abstract:**

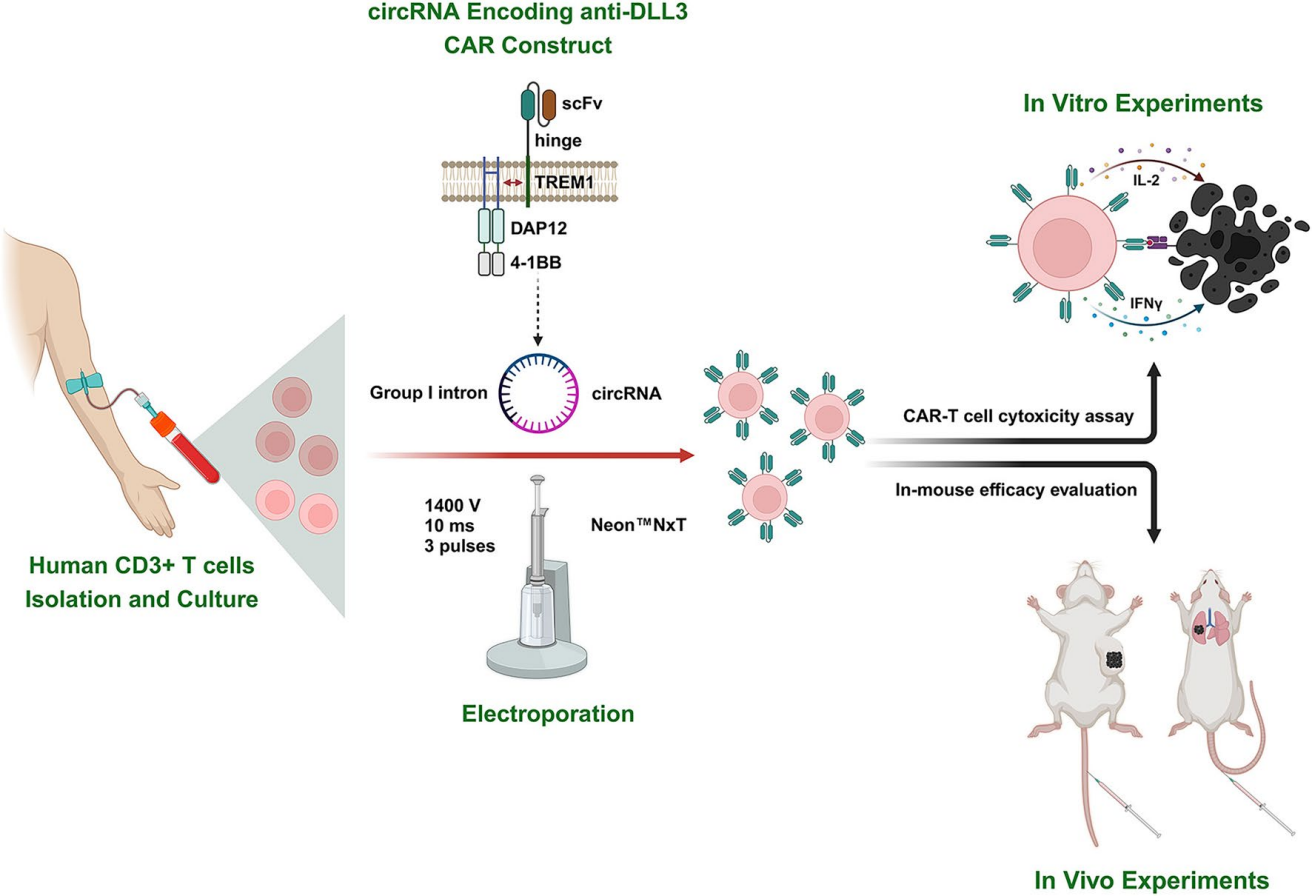

**Supplementary Information:**

The online version contains supplementary material available at 10.1186/s40164-025-00625-8.

## Introduction

The approval of two messenger RNA (mRNA)-based COVID-19 vaccines have underscored the remarkable potential of RNA-based therapeutics [1–3]. Circular RNA (circRNA) is a covalently closed single-stranded RNA molecule [4, 5]. It is reported to present notable advantages over mRNA, including enhanced stability and extended protein expression duration, which may further enhance the efficacy of RNA-based therapies [6–8]. Recently, advances in RNA structural understanding and nucleic acid technologies have enabled the synthesis of large quantities of translatable circRNA in vitro [9]. These methods include chemical synthesis [10], T4 ligase-mediated cyclization [11], and ribozyme-assisted techniques [7]. In particular, Group I intron-mediated circRNA synthesis, an optimized ribozyme-based approach, has emerged as a prominent method due to its high efficiency in generating longer circRNA products [7]. Theoretically, large-scale synthesis of circRNAs encoding target proteins can be achieved in vitro. These circRNAs can then be delivered to recipient cells, enabling therapeutic interventions for various diseases [12].

Currently, circRNA research has expanded across various applications [12]. These include vaccines [13], gene expression modulation [14], adoptive cell therapies [15], and protein replacement therapies [16]. In the domain of adoptive cell therapy, employing circRNA to construct transient Chimeric Antigen Receptor (CAR)-T cells offers a promising solution to the limitations of traditional virus-based CAR-T therapies [17]. The production of virus-based CAR-T cells involves a highly intricate process comprising several critical steps, including peripheral blood leukocyte isolation, viral vector-mediated genetic modification, CAR-T cell expansion, rigorous quality control, and reinfusion into patients [18–20]. Each of these steps must adhere strictly to Good Manufacturing Practice (GMP) standards, necessitating advanced technical expertise and specialized equipment, which poses significant challenges for implementation in standard hospital settings [21]. Moreover, the substantial costs associated with research and manufacturing create significant barriers to access for many patients who could potentially benefit from CAR-T therapy [22]. At last, traditional CAR-T approaches, which employ viral vectors to integrate CAR genes into the genome of host T cells, result in the permanent expression of CAR constructs [23]. While effective, this strategy carries the risk of long-term adverse effects, including cytokine release syndrome (CRS), neurotoxicity, and other potentially severe complications [24, 25]. These challenges highlight the pressing need to refine CAR-T therapy to improve its safety and efficacy. The use of circRNA-based CAR-T cell construction presents a promising approach to address these limitations effectively.

An efficient and reliable delivery method for introducing circRNA into primary human T cells is crucial for advancing this technology [12]. At present, the commonly used methods include electroporation and lipid nanoparticles (LNPs) [12]. While LNPs hold significant promise for future applications, their use is still under investigation due to relatively low transfection efficiency and material-related toxicity [26]. In contrast, electroporation is a well-established technique for delivering nucleic acids into primary cells including T cells, macrophages, and dendritic cells [27, 28]. Therefore, the combination of CAR construct-encoding circRNA with the electroporation technique represents a promising strategy for constructing CAR-T cells in vitro.

In this study, we compared the properties of circRNA and mRNA, confirming that circRNA exhibits superior stability and prolonged protein expression. Building on this, we optimized the electroporation conditions and employed a circRNA-electroporation strategy to engineer CAR-T cells targeting Delta-like ligand 3 (DLL3), a prominent target in small cell lung cancer (SCLC). The CAR-T cells engineered by circRNA demonstrated robust anti-tumor activity in both in vitro and in vivo models. This research not only established the feasibility of the circRNA-electroporation-based CAR-T approach but also highlighted its potential as a novel therapeutic strategy for SCLC.

## Materials and methods

### Cell lines and culture

Primary human CD3 + T cells were utilized in this study. The isolation, expansion, and culture procedures were as follows: briefly, peripheral blood mononuclear cells (PBMCs) were isolated from healthy donor using Ficoll density gradient centrifugation (Dakewe, 7922112). CD3 + T cells were then purified employing the EasySep^™^ Human CD3 Positive Selection Kit II (Stemcell, 17851). Following isolation, T cells were activated with the ImmunoCultTM Human CD3/CD28 T Cell Activator (Stemcell, 10971) and cultured in X-VIVO 15 medium (Lonza, 04-418Q) supplemented with 5% heat-inactivated fetal bovine serum (FBS) and 200 U/ml IL-2 (Stemcell, 78036.1). T cells were maintained at a density of 2 × 10^6 cells/mL in fresh culture medium. On day 12, T cells were either subjected to electroporation or cryopreserved.

Several DLL3-positive SCLC lines, including SHP-77, H69, and H524 (kindly gifted by Prof. Ming Sun, Nanjing Medical University), were utilized. Normal lung bronchial epithelial cells (BEAS-2B and 16HBE) were purchased from the China Infrastructure of Cell Line Resources. This study also utilized 293T and the lung cancer cell lines H1299 and A549, all obtained from the Institute of Biochemistry and Cell Biology, Chinese Academy of Sciences. SHP-77, H69, H524, and 16HBE cells were cultured in RPMI 1640 medium, while 293T, H1299, A549 and BEAS-2B cells were maintained in DMEM medium. All culture media were supplemented with 10% FBS and 1% penicillin/streptomycin (PS). The cells were incubated at 37°C in a humidified environment with 5% CO2 and regularly tested to confirm the absence of mycoplasma contamination.

### circRNA synthesis

The circRNA was produced using the group I intron self-splicing system as previously reported [7]. Specifically, a T7 promoter, the gene of interest (GOI) sequence, Coxsackievirus B3 (CVB3) Internal Ribosome Entry Site (IRES) elements, permuted intron–exon (PIE) constructs, and spacers were designed and cloned into a pUC57 vector to generate the plasmid (Nanjing GenScript Biotechnology Co., Ltd.). Plasmids were isolated using the TIANprep Midi Plasmid Kit (Tiangen, DP106-02). Subsequently, the plasmids were linearized using NdeI restriction endonuclease (NEB, R0111S). The linearized DNA segments were then amplified using a high-fidelity Taq enzyme kit (TOYOBO, KOD-401) to generate the GOI template. The circRNA was synthesized via in vitro transcription (IVT) using the HiScribe^™^ T7 Quick High Yield RNA Synthesis Kit (NEB, E2050S) following the manufacturer's protocol, with incubation at 37 °C for 2 h. The reaction mixtures were treated with DNase I (NEB, M0303S) at 37 °C for 10 min to remove DNA template. Additional GTP was added to a final concentration of 2 mM, and the reactions were heated at 55 °C for 15 min. Finally, the synthesized RNA was purified using the Monarch^®^ RNA Cleanup Kit (NEB, T2040L). The concentration and quality of circRNA were assessed using Thermo Scientific™ NanoDrop^™^ One spectrophotometer. To validate the production of circRNA, we employed RNase R (Novoprotein, E224) digestion on the RNA products at 37 °C for 30 min, followed by confirmation through agarose gel electrophoresis. Furthermore, circRNA was reverse transcribed into complementary DNA (cDNA) and subsequently subjected to Sanger sequencing to examine splicing junctions.

### mRNA synthesis

The mRNA template included a T7 promoter, 3' and 5' untranslated regions (UTRs), and GOI. The initial steps of mRNA synthesis were similar to those of circRNA synthesis. However, during the IVT reaction, N1-methylpseudouridine (m1Ψ, Jiangsu Synthgene Biotechnology Co., Ltd,) was used as a complete substitute for uridine. Subsequently, the mRNA precursor was subjected to DNase I treatment, followed by capping and polyadenylation reactions. For capping, mRNA cap 2′-O-methyltransferase (NEB, M0366S) and Vaccinia capping enzyme (NEB, M2080S) were used according to the manufacturer’s instructions. PolyA tail was then added to the capped linear mRNA using *E. coli* Poly(A) Polymerase (NEB, M0276S). Finally, the completed mRNA was purified using column purification.

### High performance liquid chromatography purification (HPLC)

HPLC fractionation was carried out using a 4.6 × 300 mm size exclusion column (Sepax Technologies, 215980P-4630) with a 5 µm particle size and 2000 Å pore size. The mobile phase was PBS buffer (pH 6) at a flow rate of 0.5 mL/min. RNA detection was performed via UV absorbance at 260 nm, although collection was done without UV monitoring. RNA fractions were manually collected and re-precipitated using 5 M ammonium acetate. Specifically, 0.15 volumes of 5 M ammonium acetate were added and mixed, followed by 2.5 volumes of 100% ethanol. The mixture was incubated overnight at − 80 °C. The RNA was then pelleted by centrifugation at 12,000 g for 15 min. The supernatant was carefully removed, and the RNA pellet was washed by soaking in 2.5 volumes of 70% ethanol for 2 min. The pellet was centrifuged again at 12,000 g for 5 min, and the ethanol was removed. The pellet was dried in an open tube covered with a Kimwipe for 30 min at room temperature, and then the RNA was dissolved in nuclease-free water.

### CAR construct

**Fig. 1:**
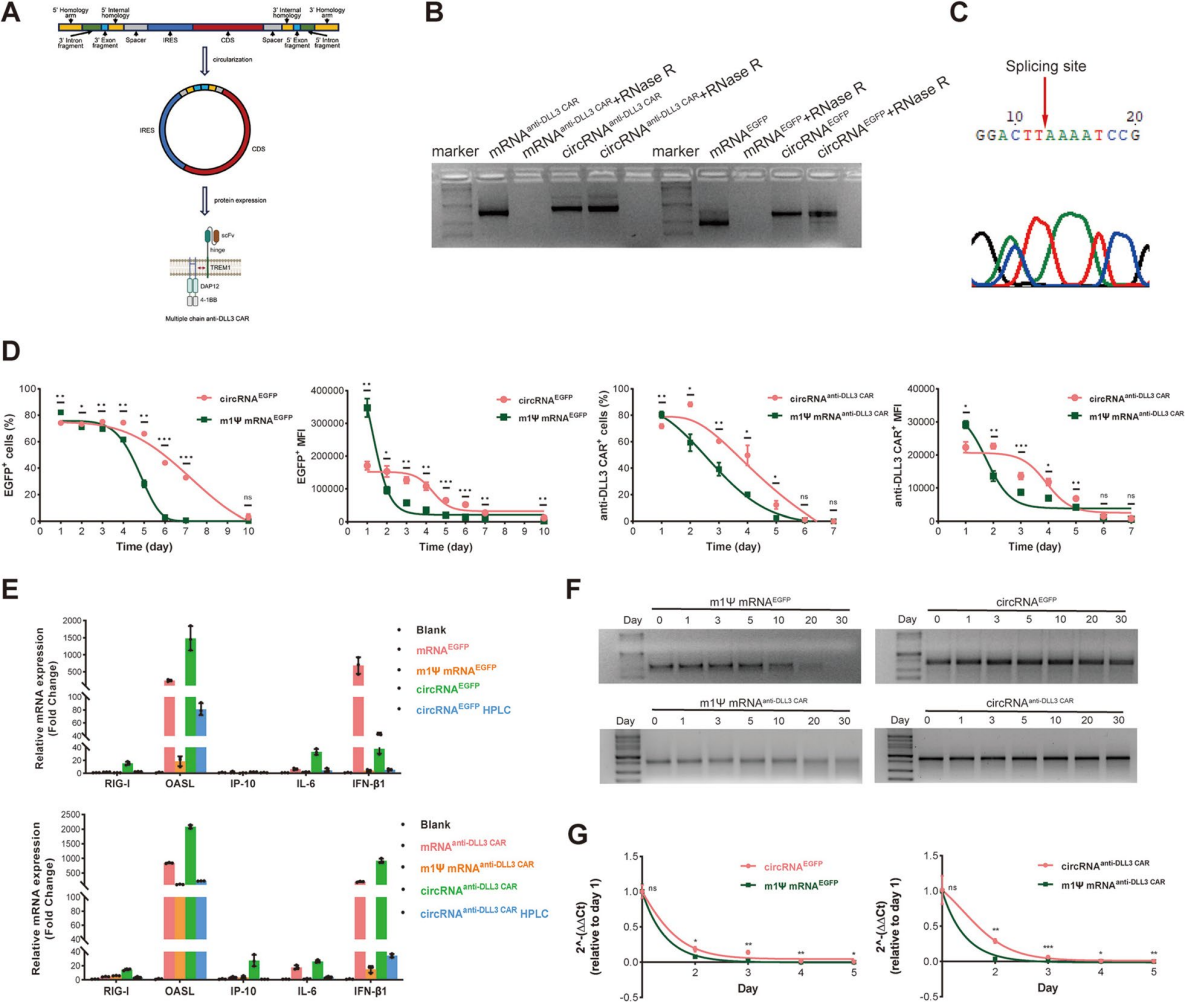
Engineering of circRNA for potent anti-DLL3 CAR protein translation. **A** Schematic representation of the construction of circRNA encoding anti-DLL3 CAR using the Orna circularization scaffold, with protein translation initiated by the CVB3 IRES sequence. **B** Agarose gel electrophoresis of RNAs treated with RNase R. **C** Sanger sequencing verification of circRNA constructs. **D** Comparative analysis of EGFP and anti-DLL3 CAR protein expression in 293T cells transfected with circRNA and mRNA. **E** Assessment of immunogenicity in A549 cells transfected with EGFP and anti-DLL3 CAR mRNAs, m1ψ mRNAs, circRNAs, and HPLC-purified circRNAs. **F** RNA stability evaluation at room temperature for 30 days. **G** Analysis of intracellular RNA stability in 293T cells transfected with circRNA and mRNA. *circRNA*, circular RNA, *mRNA* messenger RNA, *EGFP* enhanced green fluorescent protein, *CAR* chimeric antigen receptor, *DLL3* Delta-like Ligand 3, *CVB3* Coxsackievirus B3, *IRES* Internal Ribosome Entry Site, *m1ψ* N1-methylpseudouridine, *HPLC* high performance liquid chromatography

In our previous studies, we have developed an innovative multiple-chain DLL3-targeting Triggering Receptor Expressed on Myeloid Cells 1 (TREM1)/DNAX activation protein of 12kDa (DAP12) CAR structure, which included an anti-DLL3 single chain variable fragment (scFv) sequence, TREM1 hinge, DAP12 signaling domain and 4-1BB co-stimulatory domain (Fig. 1A) [29, 30]. The sequences of humanized DLL3 nanobody were obtained from a patent (US202017618642A, Nanjing Legend Biotechnology Co., Ltd.) [31], and the anti-DLL3 scFv was designed based on the variable region sequences of the heavy and light chains. Finally, the full sequence of the DLL3 targeting CAR construct was cloned into the IVT template.

### Generation of CAR-T cells

T cells were transfected using the Neon™ NxT electroporation system (ThermoFisher, Neon1) with the 100 µL kit (ThermoFisher, N10096) according to the manufacturer's instructions. Specifically, T cells were washed twice with pre-warmed PBS, and 3–4 × 10^6 T cells were resuspended in 100 µL of Buffer R containing 15–20 µg of circRNA. The cell suspension was then transferred to the electroporation tip. Electroporation was performed at 1400V, with a pulse width of 10 ms and 3 pulses. After electroporation, the cells were transferred to pre-warmed RPMI 1640 medium supplemented with 10% heat-inactivated FBS, without PS.

### Flow cytometry

BD FACSCalibur flow cytometry was utilized in this study. After trypsin digestion, cells were washed twice with PBS and resuspended in 100 µL PBS containing 1% heat-inactivated FBS. Fluorescently labeled antibodies were added according to the manufacturer’s instructions, followed by incubation at 4 °C for 30 min. The cells were then washed twice with PBS and finally resuspended in 500 µL PBS for analysis. APC anti-human CD3 antibody (Proteintech, APC-65133) was used to label CD3 + T cells. PE MonoRab^™^ Rabbit Anti-scFv Cocktail (Nanjing GenScript Biotechnology Co., Ltd., A02285) were employed to assess CAR expression. DLL3 expression on target cells was evaluated using an anti-DLL3 antibody (Abcam, ab229902) with PE donkey anti-rabbit IgG (Biolegend, 406421). Cell viability was determined using the 7-AAD viability staining solution (Biolegend, 420403) and the annexin V/PI cell apoptosis detection kit (Servicebio, G1511-50T). The activation status of CAR-T cells was assessed with FITC anti-human CD69 antibody (Biolegend, 310903) and PerCP anti-human CD25 antibody (Biolegend, 356131). The phenotype of CAR-T cells after coculture was identified using PE anti-human CD45RO antibody (Biolegend, 304205), PE/Cyanine7 anti-human CD45RA antibody (Biolegend, 304125) and APC anti-human CCR7 antibody (Biolegend, 353213). Data analysis was performed using FlowJo software, which could determine the positive rate and median fluorescence intensity (MFI) of the cell populations.

### In vitro cytotoxicity assay

To monitor cytotoxic effects in co-culture assays, SHP-77 and H69 cells were genetically modified to stably express GFP (GFP SHP-77 and GFP H69, kindly gifted by Prof. Ming Sun, Nanjing Medical University). 30,000 target cells were seeded in a 96-well plate and co-cultured with CAR-T cells at effector-to-target (ET) ratios of 0.25:1, 0.5:1, 1:1, 2:1, 5:1, and 10:1 for 24 h. Following the incubation, the proportion of GFP-positive cells was analyzed using fluorescence microscopy and flow cytometry. Total cellular RNA was extracted for transcriptional analysis of cytotoxic cytokines.

### Enzyme linked immunosorbent assay (ELISA)

The secretion levels of cytotoxic cytokines were evaluated using Human IL-2 ELISA kit (NeoBioscience, EHC003), Human IFN-γ ELISA kit (NeoBioscience, EHC102g), and Human TNF-α ELISA kit (NeoBioscience, EHC103g). Assays were conducted following the manufacturer’s protocols. Briefly, co-culture supernatants were centrifuged and diluted tenfold before being added, along with standards, to pre-coated 96-well ELISA plates. Plates were incubated at 37 °C for 90 min. After five washes to remove unbound proteins, biotinylated antibodies were added and incubated at 37 °C for 60 min in the dark. Following another five washes, enzyme conjugate working solution was added and incubated at 37 °C for 30 min, followed by a final series of five washes. The substrate solution was then added, and plates were incubated at 37 °C for 15 min. The reaction was terminated with stop solution, and absorbance was measured at optical density (OD) 450 using a microplate reader (BioTek Synergy LX).

### Cell proliferation assay

The Cell Counting Kit-8 (CCK-8, Biosharp, BS350A) assay was utilized to evaluate T cell proliferation. T Cells were seeded in a 96-well plate at a density of 3 × 10^4 cells per well in 100 μL of culture medium. After incubating for 24 h, 10 μL of CCK-8 solution was added to each well. Cells were then incubated for an additional 2 h at 37 °C. The absorbance of each well was measured at 450 nm using a microplate reader (BioTek Synergy LX). The OD values were proportional to the number of viable cells, and cell proliferation was determined by comparing the OD values of treated samples to controls. The Carboxyfluorescein Succinimidyl Ester (CFSE) assay was also employed to assess cell proliferation by tracking cell division. The CFSE working solution, prepared at an appropriate concentration, is added to 1 mL of T cell suspension resuspended in PBS, containing 10–100 × 10^6 cells. Incubate the cells in the CFSE solution at room temperature or 37 °C for 20 min, ensuring that the cells are protected from light during this period. To halt the staining process, add an amount of cell culture medium containing 10% FBS equal to five times the original volume of the CFSE solution. After adding the quenching medium, centrifuge the cells to pellet them, then resuspend in pre-warmed cell culture medium. After a 4-day incubation period, the CFSE-labeled cells are analyzed by flow cytometry to assess changes in GFP intensity and the proportion of cells with varying levels of fluorescence.

### Real-time cell analyzer (RTCA)

RTCA (Agilent, xCELLigence RTCA TP) was employed to monitor and quantify cell proliferation and viability continuously. Cells were cultured and prepared by trypsinization, counting, and resuspending in fresh medium before seeding into the wells of an RTCA E-Plate at a concentration of 5 × 10^3 target cells per well. Following cell adhesion, the baseline impedance was recorded to establish initial cell index values. After 24 h of target cell incubation, CAR-T cells are added, and the system continues to monitor the incubation system for up to 96 h. This impedance-based technology reflects changes in cell adhesion, proliferation, and morphology in real-time, converting impedance data into cell index values that are plotted as growth curves. Experiments were conducted in triplicate to ensure reproducibility.

### Immunohistochemistry staining

Immunostaining was conducted on formalin-fixed, paraffin-embedded tissues from both patients and mouse models. The specimens were first dewaxed and rehydrated, undergoing three immersions in xylene, followed by a series of immersions in varying concentrations of ethanol. They were then rinsed with citrate buffer and Triton X-100 (Solarbio, T8200), and washed in PBS three times for 3 min each. Following this, heat-induced epitope retrieval was performed by placing the specimens in a 95°C water bath for 5 min, after which they were cooled to room temperature. The samples were rinsed again and blocked with animal-free blocking solution (Cell signaling Technology, 15,019) at room temperature for 15 min, then incubated with the primary anti-DLL3 antibody (Abcam, ab229902, 1:1000 dilution) overnight in 4°C. Subsequently, the slides were incubated with a horseradish peroxidase (HRP)-labeled secondary antibody (Beyotime, A0208, 1:50 dilution) for 1 h at room temperature, stained with diaminobenzidine (Solarbio, DA1015), and observed under a microscope.

### Immunofluorescence staining

To investigate DLL3 localization, we performed immunofluorescence assays using H69, H524, SHP-77, BEAS-2B, and 16HBE cells. The cells were seeded into 24-well plates, fixed with 4% paraformaldehyde for 30 min, and then permeabilized with 0.4% Triton X-100 (Solarbio, IT9100) for 30 min. Next, the cells were blocked with 2% bovine serum albumin (BSA) for 30 min. After blocking, the cells were incubated overnight at 4°C with primary antibodies against DLL3 (Abcam, ab229902, 1:100 dilution). In the dark, the cells were then incubated with goat anti-rabbit IgG H&L (AF647) antibodies for 30 min, followed by nuclear staining with DAPI. Finally, the samples were sealed with an anti-fade mounting medium (Solarbio, S2100), and images were acquired using a confocal microscope (Nikon, A1RSi + N-STORM).

### Gel electrophoresis

RNA and DNA products were analyzed using 2% agarose gel electrophoresis with 1 × TAE running buffer containing 1 × GelRed (Aoqing, 218251122). Samples were mixed with 2 × loading buffer and subjected to electrophoresis at 120 V for 30 min. An ssRNA Ladder (NEB, N0362S) served as the standard. Bands were visualized under UV irradiation (Bltlux, GelView 6000Plus).

### Western blot (WB)

Proteins were extracted from cells using Western and IP lysis buffer (Beyotime, P0013) containing 1% protease inhibitor mixture (Solarbio, P6730) on ice for 30 min, followed by centrifugation at 12,000 g for 20 min at 4 °C. The supernatants were collected into fresh 1.5 mL tubes, and protein concentrations were measured with a bicinchoninic acid assay kit (LABLEAD, B5001). The protein samples were then separated on FuturePAGE^™^ 4–12% 12-well gels (ACE Biotechnology, ET12412Gel) at 150V for 50 min and transferred to PVDF membranes using Trans-Blot^®^ Turbo™ Universal Protein Transfer System (BIO-RAD). The membranes were blocked with 5% BSA in Tris-buffered saline with Tween-20 (TBST) (Solarbio, T1082), followed by overnight incubation at 4°C with primary antibodies. After three washes with TBST, the membranes were incubated with HRP-conjugated secondary antibodies for 2 h at room temperature. Finally, the membranes were developed using a chemiluminescent HRP substrate (NCM Biotech, P10300) and imaged with an electrogenerated chemiluminescence system (Btlux, GelView 6000Plus). The antibodies are listed as follows: anti-DLL3 (abcam, ab229902), anti-GFP (MBL, 598), anti-DAP12 (Signalway Antibody, 49944), anti-actin (Proteintech, 66009–1-Ig), anti-GAPDH (Proteintech, 60004–1-Ig), anti-tubulin (Proteintech, 66031–1-Ig), anti-scFv (Genscript Biotech, A02282).

### Real time polymerase chain reaction (PCR)

Cellular RNA was extracted using the FastPure Cell/Tissue Total RNA Isolation Kit V2 (Vazyme, RC112-01) following the manufacturer's protocol. The quality of the extracted RNA was assessed with a Thermo Scientific™ NanoDrop™ One spectrophotometer. RNA was then reverse-transcribed into cDNA using the PrimeScript^™^ Master Mix (TaKaRa, RR037A). Relative quantification of RNA expression was performed using SYBR Green I dye (Vazyme, Q111-02), with qPCR conducted on an Agilent AriaDx Real-Time PCR System. The primers used in this study are listed in Supplementary Table 1.

### Xenograft model and treatments

Six to eight-week-old male NCG (NOD/ShiLtJGpt-Prkdc^em26Cd52^Il2rg^em26Cd22^/Gpt) mice, obtained from the GemPharmatech (T001475), were used for all xenograft experiments. The mice were maintained under SPF-grade conditions in the animal facility at the Department of Laboratory Animal Science, Peking University Health Science Center. All animal experiments adhered strictly to the compliance standards set by the Institutional Animal Care and Use Committee. For the subcutaneous tumor model, 2 × 10^6 luciferase-labeled SHP-77 cells in 100 μL of PBS were injected subcutaneously into mice, and tumors were allowed to develop for approximately 14 days. The experimental group received tail vein injections of 2 × 10^6 CAR-T cells in 100 μL of PBS every four days, for a total of five treatments. The control group received either 100 μL of PBS or the same number of normal T cells. Tumor size was measured every three days using calipers, with volume calculated as volume (mm^3^) = length × width^2^ / 2. Mice were weighed every three days. Tumor size was also assessed every seven days via bioluminescence imaging (Btlux, AniView100). On day 35, the mice were euthanized, and their major organs were collected for H&E staining. For orthotopic lung tumor model, under complete anesthesia, a small incision was made on the left side of the mice to access the thoracic cavity. Subsequently, 1 × 10^6 luciferase-labeled SHP-77 cells suspended in 50 μL of PBS were injected directly into the lung parenchyma using a fine-gauge needle. The incision was then closed with appropriate sutures to prevent leakage and contamination. Tumors were allowed to grow for approximately 10 days. The experimental group received tail vein injections of 2 × 10^6 CAR-T cells in 100 μL of PBS every four days, totaling eight treatments. The control group was also administered either 100 μL of PBS or an equivalent number of normal T cells. Mice were weighed every three days. Tumor growth was monitored weekly using bioluminescence imaging. On day 35, the mice were euthanized, and their major organs were harvested for H&E staining.

### Bioinformatics analysis

The bioinformatics analysis was performed by Annoroad Gene Technology (Beijing) Co., Ltd. The data has been uploaded to the NCBI Sequence Read Archive database under the accession number SRR32080050.

### Statistical analysis

Experimental data were analyzed using GraphPad Prism version 9.0 and ImageJ software. Group differences were evaluated using unpaired, two-tailed Student’s t-tests or two-way ANOVA. Statistical significance was determined by a p-value of less than 0.05. Significance levels were defined as *p < 0.05, **p < 0.01, and ***p < 0.001. Data are presented as means ± standard deviation (SD). All experiments were performed with at least three replicates per group (n ≥ 3).

## Results

### Engineering circRNA for potent anti-DLL3 CAR protein translation

In this study, we constructed circRNAs encoding enhanced green fluorescent protein (EGFP) and anti-DLL3 CAR using the Orna circularization scaffold [7], with protein translation initiated by the CVB3 IRES sequence (Fig. 1A). By analyzing public Gene Expression Omnibus (GEO, GSE149507) and Cancer Cell Line Encyclopedia (CCLE) databases, we preliminarily demonstrated that DLL3 is highly expressed in SCLC tissues (Figure S1A) and cell lines (Figure S1B). Immunohistochemical evaluation of 30 SCLC tissues and 10 normal lung tissues further confirmed high DLL3 expression in SCLC tissues and very low expression in normal lung tissues (Figure S1C). Subsequently, we validated high DLL3 expression in three SCLC cell lines (H69, H524, and SHP-77) using WB (Figure S1D), RT-qPCR (Figure S1E), and flow cytometry (Figure S1F), respectively. Normal lung cell lines (BEAS-2B and 16HBE) showed no DLL3 expression (Figure S1D-F). Finally, immunofluorescence staining confirmed the membrane localization of DLL3 in SCLC cells (Figure S1G). RNase R treatment of EGFP/anti-DLL3 CAR encoding-mRNA and circRNA revealed that mRNA was degraded, while circRNA remained intact due to its closed-loop structure, which confers resistance to RNase R (Fig. 1B). Then, circRNA was reverse transcribed, and the resulting cDNA was amplified using primers spanning the splicing junction, and sanger sequencing confirmed the circularization of the RNA constructs (Fig. 1C). Furthermore, after transfecting the EGFP/anti-DLL3 CAR encoding-circRNAs into 293T and H1299 cells, we demonstrated its protein expression capability through visible fluorescence, flow cytometry, and WB analysis (Figure S2A). Reverse-phase HPLC was employed to purify the circRNA products, and analysis of the chromatogram indicated that the peak in the latter part corresponded to the intended circRNA (Figure S2B).

### Comparing circRNA and mRNA: expression, stability, and immune response

We transfected 293T cells with equimolar quantities of circRNAs and mRNAs encoding EGFP and anti-DLL3 CAR, and monitored target protein expression daily using flow cytometry to assess the percentage of positive cells and the MFI. Results indicated that mRNA-expressed EGFP peaked on day 1 and was higher than circRNA, but its expression rapidly declined thereafter, nearing zero by day 7. In contrast, circRNA-expressed EGFP, while initially lower than mRNA, remained higher over subsequent days, sustaining approximately 30% positivity by day 7. Similar trends were also observed for anti-DLL3 CAR expression, with circRNA demonstrating a longer duration of target protein expression compared to mRNA (Fig. 1D). Similar experiments have been conducted in the H1299 cell line yielded comparable results, reinforcing our findings (Figure S2C). To evaluate immunogenicity, we transfected A549 cells with EGFP and anti-DLL3 CAR encoding mRNAs, m1ψ mRNAs, circRNAs, and HPLC-purified circRNAs. After 6 h, we measured several inflammatory cytokines and immune regulators (RIG-I, OASL, IP-10, IL-6, and IFN-β1) in total RNA extracts. The results showed that HPLC purification notably reduced circRNA's immunogenicity, making it comparable to m1ψ mRNA in most markers (Fig. 1E). Similar results were also observed in 293T and H1299 cell lines (Figure S2D). Then, we assessed the stability of circRNA and mRNA by incubating both types at room temperature for 1, 3, 5, 10, 20, and 30 days, followed by gel electrophoresis to detect degradation. Results showed that EGFP and anti-DLL3 CAR encoding-mRNA showed significant degradation after 20 days, whereas circRNA remained stable (Fig. 1F). Additionally, we evaluated RNA stability in various FBS gradients (0%, 0.1%, 0.2%, 0.3%, 0.4%, and 0.5%), and we found that mRNA began to degrade at 0.1% FBS, while circRNA remained stable until 0.3% FBS (Figure S2E). Finally, to investigate the intracellular stability of circRNA, we transfected 293T cells with circRNA and mRNA, and extracted RNA daily. Using junction and CDS primers for amplification, with day 1 as the reference, the results indicated that circRNA demonstrated greater stability within the cells compared to mRNA (Fig. 1G). Similar experiments conducted in the H1299 cell line yielded consistent results (Figure S2F). These results showed that, compared with mRNA, circRNA has enhanced stability, lower immunogenicity and extended protein expression duration.

### Optimization of electroporation conditions for circRNA expression in human primary T Cells

**Fig. 2:**
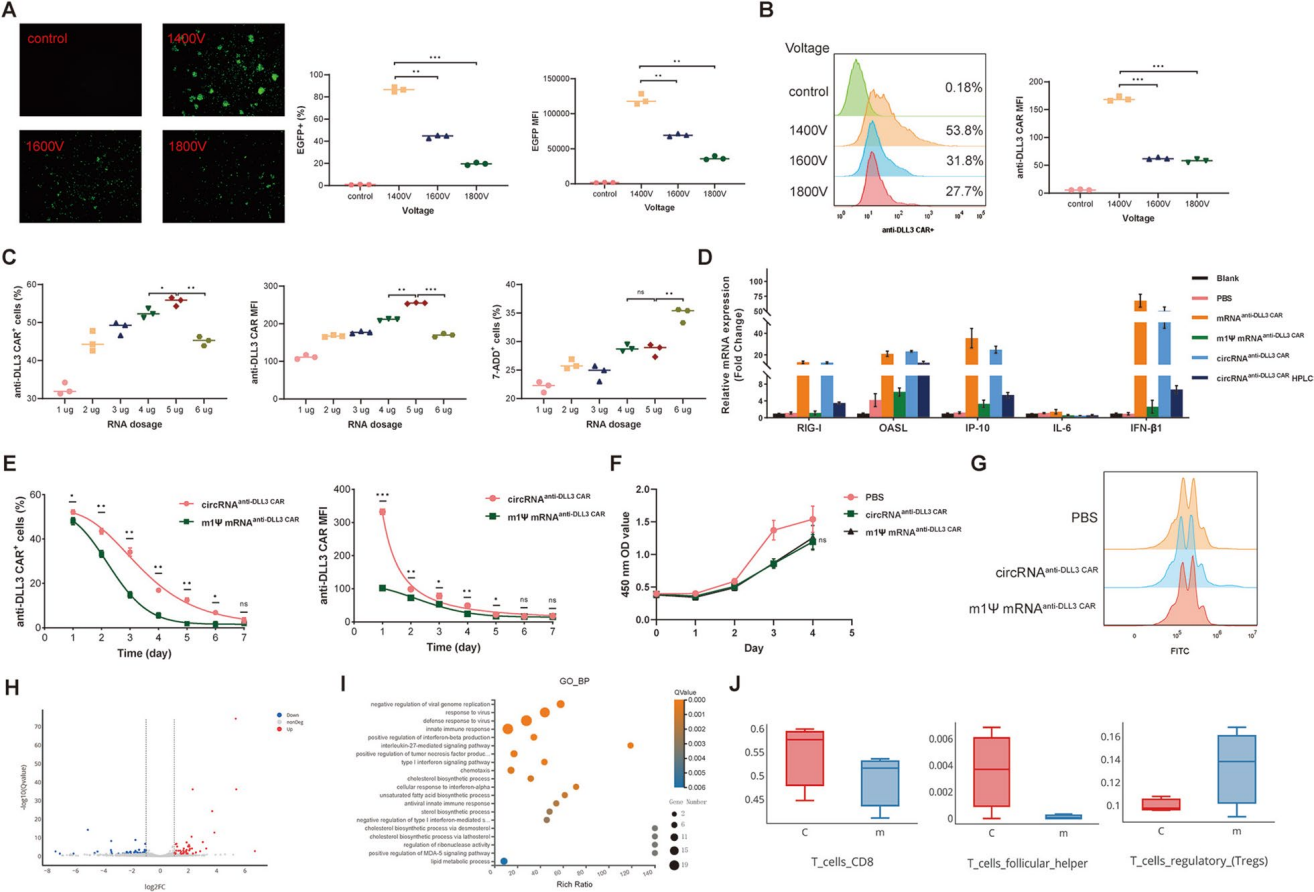
Optimization of electroporation conditions for circRNA expression in Human CD3 + T cells. **A** Comparison of EGFP expression in T cells electroporated with circRNA at different voltages (1400V, 1600V, and 1800V). **B** Flow cytometry analysis of anti-DLL3 CAR expression at varying electroporation voltages. **C** Evaluation of CAR expression and apoptosis rates in T cells electroporated with various concentrations of circRNA (1–6 µg/million cells). **D** Assessment of immunogenicity in circRNA and mRNA-transfected T cells under optimized electroporation conditions. **E** Assessment of CAR expression duration in circRNA and mRNA-transfected T cells. **F** Evaluation of cell proliferation in circRNA and mRNA-transfected T cells using the CCK-8 assay. **G** Evaluation of cell proliferation in circRNA and mRNA-transfected T cells using the CFSE assay. *circRNA* circular RNA, *mRNA* messenger RNA, *EGFP* enhanced green fluorescent protein, *CAR-T* chimeric antigen receptor T cell, *DLL3* Delta-like Ligand 3, *CCK-8* Cell Counting Kit-8, *CFSE* Carboxyfluorescein Succinimidyl Ester

The Neon™ NxT electroporation system was employed to transfect circRNA into human CD3 + T cells. We first investigated the expression efficiency of circRNA under different voltages (1400V, 1600V, and 1800V). After electroporating T cells with EGFP-encoding circRNA, the 1400V condition visibly produced the highest number of EGFP-expressing T cells. Flow cytometry analysis confirmed that 1400V resulted in an EGFP-positive T cell population exceeding 80%, significantly higher than the 1600V and 1800V conditions, with similar trends observed in the MFI data (Fig. 2A). We also evaluated anti-DLL3 CAR expression under these three voltage conditions, further confirming that 1400V is the optimal voltage for achieving the highest efficacy (Fig. 2B). Next, we explored the optimal dosage of circRNA by testing six concentration gradients (1 µg/million cells, 2 µg/million cells, 3 µg/million cells, 4 µg/million cells, 5 µg/million cells, and 6 µg/million cells). Flow cytometry was used to assess CAR expression rate, MFI, and apoptosis levels. The results indicated that at 5 µg/million cells, CAR expression was maximized (approximately 55.0%) while apoptosis remained within an acceptable range (Fig. 2C). We have also explored the optimal electroporation voltage and dose for mRNA. The results indicated that 1400V is the optimal electroporation voltage, and 4 µg/million cells is the optimal dose (Figure S3). Under the optimal voltage and dosage, we further compared the immunogenicity, expression duration, and impact on cell proliferation between circRNA and mRNA. The results indicated that electroporation alone did not affect the expression levels of inflammatory cytokines and immune regulators, and HPLC-purified circRNA exhibited significantly reduced immunogenicity compared to unpurified circRNA (Fig. 2D). Consistent with previous findings, circRNA-based CAR expression in T cells was significantly more sustained than mRNA. Notably, on day 1, CAR expression was already higher in circRNA-transfected cells than in those transfected with mRNA (Fig. 2E). CCK-8 assays showed no difference in cell viability between circRNA and mRNA electroporation (Fig. 2F), which was further confirmed by CFSE assays (Fig. 2G). We conducted a comparative transcriptomic analysis between circRNA^anti−DLL3 CAR−T^ and mRNA^anti−DLL3 CAR−T^ cells, revealing 42 upregulated and 61 downregulated differentially expressed genes (DEGs) (Fig. 2H). Gene enrichment analysis showed that these DEGs were predominantly enriched in effector cytokine signaling pathways, such as IFN-β and TNF pathways (Fig. 2I). Further examination of the immune microenvironment differences between the two groups demonstrated a higher proportion of CD8 + T cells and follicular helper T cells in the circRNA^anti−DLL3 CAR−T^ group, with a lower percentage of regulatory T (Treg) cells (Fig. 2J). These findings demonstrated the feasibility of using RNA-electroporation to generate CAR-T cells, and highlighted that circRNA-based CAR-T cells outperform mRNA-based CAR-T cells in terms of sustained CAR expression and enhanced cytotoxic activity.

### Efficient killing of DLL3-positive SCLC cells by circRNA-based CAR-T cells

**Fig. 3:**
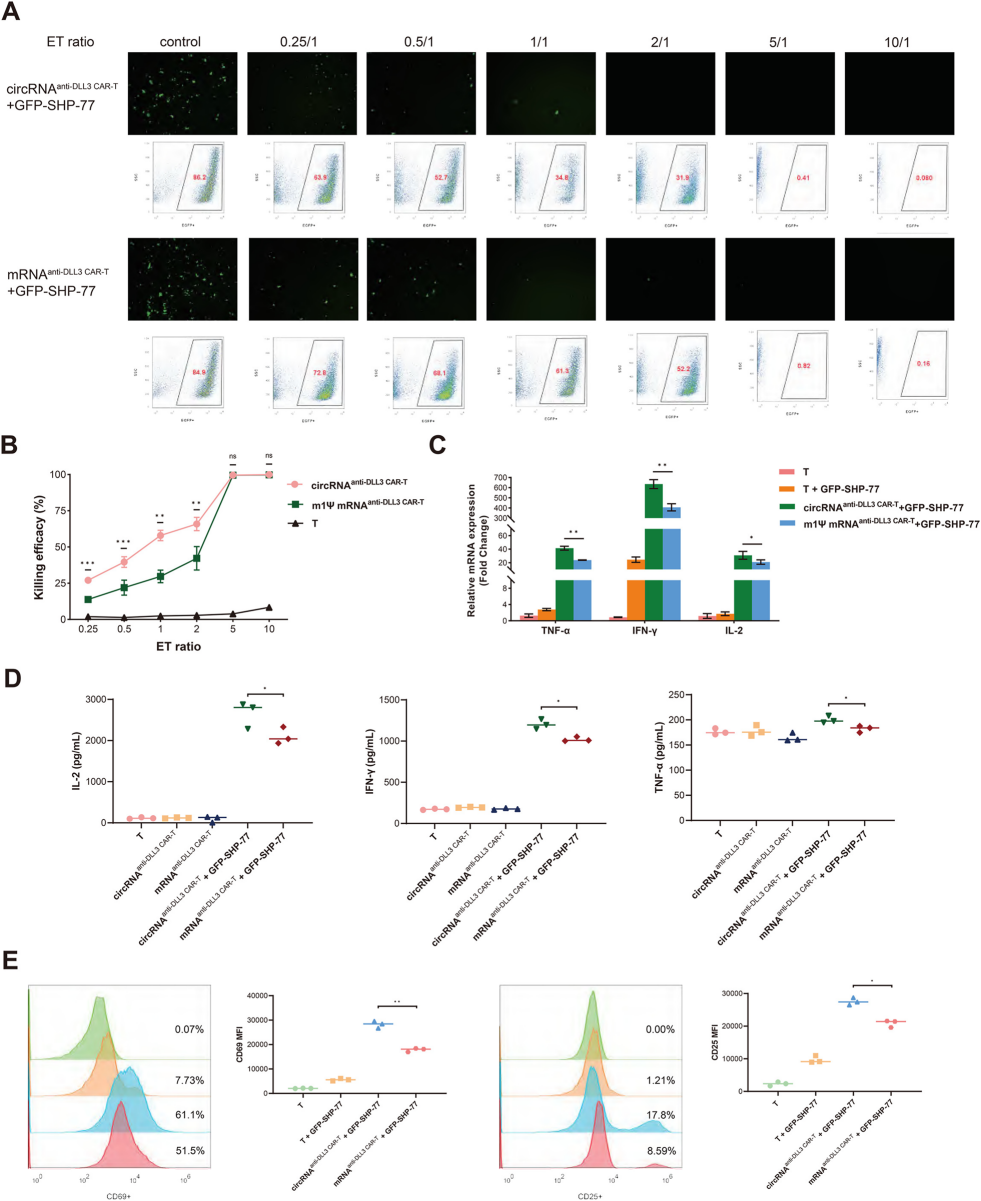
Efficient killing of DLL3-positive SCLC cells by circRNA-based CAR-T cells. **A** Fluorescence microscopy of co-cultures of DLL3-positive GFP-expressing SCLC cell lines (GFP-SHP-77 and GFP-H69) with circRNA-based anti-DLL3 CAR-T cells at various ET ratios. **B** Flow cytometry analysis of the killing efficiency of circRNA^anti−DLL3 CAR−T cells^ compared to mRNA^anti−DLL3 CAR−T cells^. **C** qPCR analysis of cytotoxic cytokine expression in the co-culture system. **D** ELISA analysis of cytokine levels in co-culture supernatants. **E** Comparison of CD69 + and CD25 + T cell activation proportions between circRNA^anti−DLL3 CAR−T^ and mRNA^anti−DLL3 CAR−T^ groups. *circRNA* circular RNA, *mRNA* messenger RNA, *GFP* green fluorescent protein, *CAR-T* chimeric antigen receptor T cell, *DLL3* Delta-like Ligand 3, *ET* effector-to-target, *qPCR* real time fluorescence quantitative polymerase chain reaction, *ELISA* enzyme linked immunosorbent assay, *SCLC* small cell lung cancer

To evaluate the cytotoxic efficacy of anti-DLL3 CAR-T cells constructed by circRNA and electroporation, we co-cultured them with two DLL3-positive, GFP-expressing SCLC cell lines, GFP-SHP-77 and GFP-H69. The co-cultures were performed at various ET ratios (0.25:1, 0.5:1, 1:1, 2:1, 5:1, and 10:1) for 24 h, and untreated T cells were included as control groups. Under fluorescence microscopy, an increasing ET ratio led to a noticeable reduction in GFP-positive cells in both the circRNA^anti−DLL3 CAR−T^ and mRNA^anti−DLL3 CAR−T^ groups, with no GFP-positive cells observed at ratios of 5:1 and 10:1 (Fig. 3A). Flow cytometry revealed that circRNA^anti−DLL3 CAR−T cells^ exhibited significantly higher cytotoxicity compared to mRNA^anti−DLL3 CAR−T cells^ at ET ratios of 0.25:1, 0.5:1, 1:1, and 2:1 (Fig. 3B). circRNA^anti−DLL3 CAR−T cells^ achieved nearly 60% killing efficiency at a 1:1 ratio (Fig. 3B). Subsequently, we extracted total RNA from the co-culture system and performed qPCR to analyze the expression levels of three key effector cytokines (TNF-α, IFN-γ, and IL-2). The results showed that the circRNA^anti−DLL3 CAR−T^ group had markedly higher levels of TNF-α, IFN-γ, and IL-2 than the mRNA^anti−DLL3 CAR−T^ group (Fig. 3C). ELISA analysis of the co-culture supernatants revealed significantly higher concentrations of IL-2 and IFN-γ in the circRNA^anti−DLL3 CAR−T^ group compared to the mRNA^anti−DLL3 CAR−T^ group, with TNF-α levels slightly elevated in the circRNA^anti−DLL3 CAR−T^ group (Fig. 3D). In addition, we have also compared the proportions of activated CD69 + and CD25 + T cells between the two groups. The circRNA^anti−DLL3 CAR−T^ group showed a higher percentage of activated CD69 + T cells (61.1%) compared to the mRNA^anti−DLL3 CAR−T^ group (51.5%), and a higher percentage of activated CD25 + T cells (17.8%) compared to the mRNA^anti−DLL3 CAR−T^ group (8.59%) (Fig. 3E). At last, we have further assessed the distribution of central memory T cell (Tcm), effector memory T cell (Tem) and stem cell-like memory T cell (Tscm) subsets in the cocultured T cells. Compared to the other groups, we observed that the circRNA^anti−DLL3 CAR−T^ group exhibited the highest proportion of Tcm, whereas the proportion of Tem was comparatively lower (Figure S4A). Additionally, no significant difference in the proportion of Tscm was observed between the experimental and control groups (Figure S4B). We replicated these experiments in the GFP-H69 cell line and obtained similar results (Figure S5 and Figure S6). These findings indicated that anti-DLL3 CAR-T cells constructed by circRNA and electroporation can effectively kill DLL3-expressing SCLC cells.

### Assessment of off-target effects in anti-DLL3 CAR-T cells

**Fig. 4:**
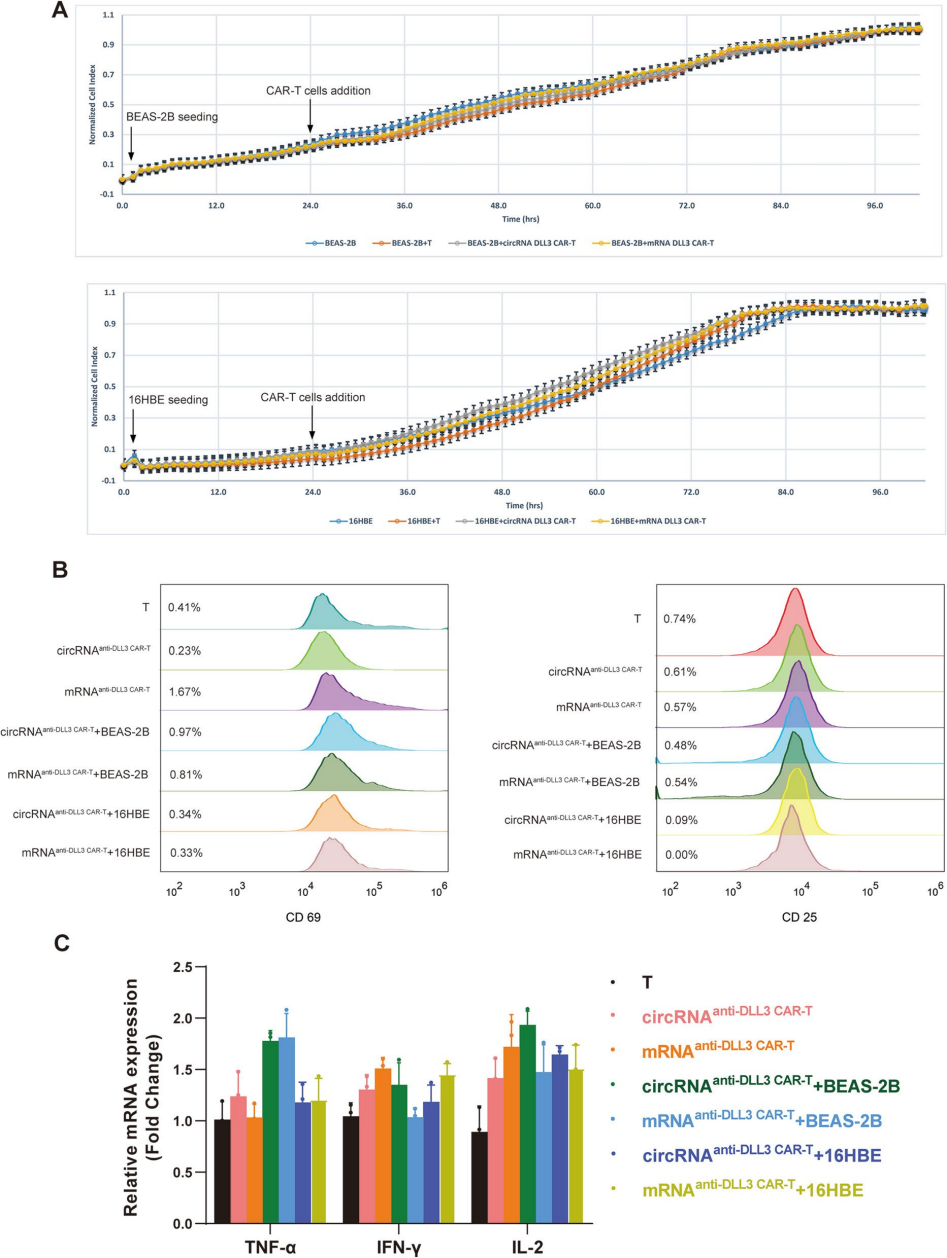
Assessment of off-target effects in anti-DLL3 CAR-T cells. **A** RTCA monitoring of the proliferation of DLL3-negative normal lung cell lines (BEAS-2B and 16HBE) co-cultured with circRNA^anti−DLL3 CAR−T^ and mRNA^anti−DLL3 CAR−T^. **B** Analysis of CAR-T cell activation markers (CD69 + and CD25 +) in the co-culture system. **C** qPCR analysis of cytokine expression in the co-culture system. *circRNA* circular RNA, *CAR-T* chimeric antigen receptor T cell, *DLL3* Delta-like Ligand 3, *ET* effector-to-target, *qPCR* real time fluorescence quantitative polymerase chain reaction, *RTCA* Real-time cell analyzer

To investigate off-target effects of anti-DLL3 CAR-T cells, we co-cultured CAR-T cells with DLL3-negative normal lung cell lines BEAS-2B and 16HBE at an ET ratio of 1:1. RTCA monitoring of BEAS-2B and 16HBE cell growth over 96 h showed no significant impact on proliferation by CAR-T cells (Fig. 4A). Similarly, assessment of CAR-T cell activation revealed no significant differences in the proportions of activated CD69 + and CD25 + T cells between CAR-T and control groups (Fig. 4B). Finally, qPCR analysis of total RNA from the co-culture system showed only slight increases in TNF-α, IFN-γ, and IL-2 levels in the CAR-T cell group (Fig. 4C). These results indicated that circRNA-engineered CAR-T cells exhibit no off-target effects, confirming their safety.

### Potent efficacy of CAR-T cells in mouse subcutaneous tumor model

**Fig. 5:**
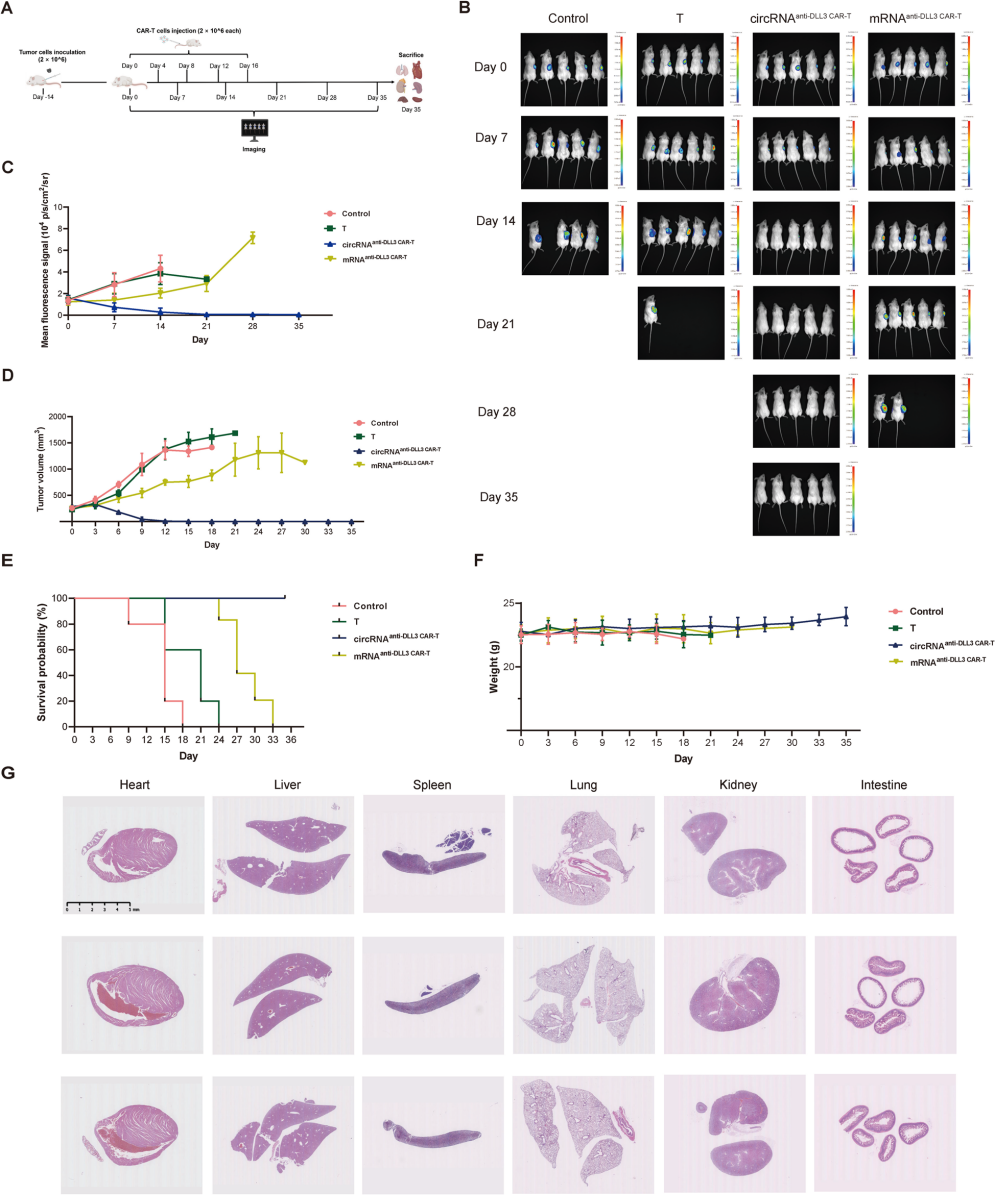
Potent efficacy of circRNA-engineered CAR-T cells in a mouse subcutaneous tumor model. **A** Experimental design for evaluating CAR-T cell efficacy in a mouse subcutaneous tumor model. 2 × 10^6 luciferase-labeled SHP-77 cells inoculated subcutaneously into mice, and tumors were allowed to develop for approximately 14 days. The experimental group received tail vein injections of 2 × 10^6 CAR-T cells every four days, for a total of five treatments. The control group received either PBS or the same number of normal T cells. **B** Bioluminescence imaging of tumor growth in mice treated with circRNA^anti−DLL3 CAR−T cells^, mRNA.^anti−DLL3 CAR−T cells^, and controls. **C** Survival analysis of mice treated with CAR-T cells. **D** Tumor volume measurements in CAR-T treated mice. **E** Weight monitoring of CAR-T treated mice. **F** Histological analysis of vital organs from CAR-T treated mice. *circRNA*,circular RNA, *CAR-T* chimeric antigen receptor T cell, *DLL3* Delta-like Ligand 3

Fourteen days after successful subcutaneous tumor establishment, in both the experimental and control groups, 2 × 10^6 CAR-T cells and 2 × 10^6 normal T cells were administered via tail vein injection on Days 0, 4, 8, 12, and 16. Tumor size was measured with calipers every three days, and bioluminescence imaging was performed weekly (Fig. 5A). Bioluminescence imaging revealed that CAR-T cells engineered by circRNA significantly inhibited tumor growth compared to the control T cells and mRNA-engineered CAR-T cells. By Day 21, tumors were undetectable and all mice survived in the group treated with circRNA-engineered CAR-T cells. In contrast, mRNA-engineered CAR-T cells only delayed tumor growth, with all mice ultimately died at day 33 (Figs. 5B and 5C). Tumor volume measurements supported the bioluminescence imaging results (Fig. 5D). Survival analysis showed that while both CAR-T groups extended mouse survival compared to the control group, all mice in the circRNA^anti−DLL3 CAR−T^ group were alive at the end of the study, whereas mice in the mRNA^anti−DLL3 CAR−T^ group began dying on Day 21, with all mice dead by Day 33. No significant weight loss (Fig. 5F) or organ necrosis (Fig. 5G) was observed in the CAR-T treated mice. Our results indicated that anti-DLL3 CAR-T cells engineered by circRNA and electroporation show excellent anti-tumor efficacy in the SCLC subcutaneous tumor model.

### Evaluation of CAR-T Cell efficacy in mouse lung orthotopic tumor model

**Fig. 6:**
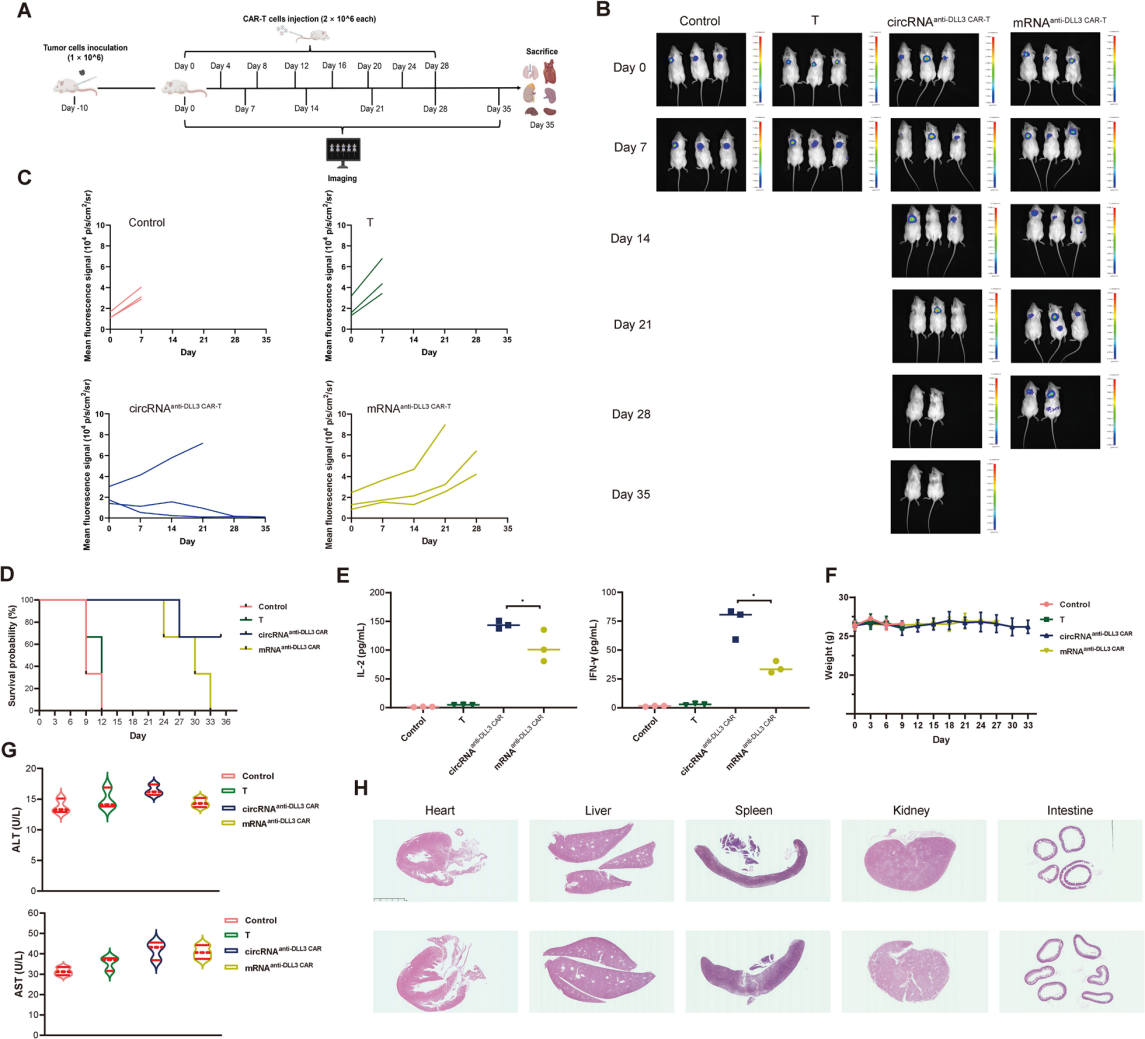
Evaluation of circRNA-engineered CAR-T cell efficacy in a mouse lung orthotopic tumor model. **A** Experimental design for evaluating CAR-T cell efficacy in a mouse lung orthotopic tumor model. 1 × 10^6 luciferase-labeled SHP-77 cells inoculated subcutaneously into mice, and tumors were allowed to develop for approximately 14 days. The experimental group received tail vein injections of 2 × 10^6 CAR-T cells every four days, for a total of five treatments. The control group received either PBS or the same number of normal T cells. **B** Bioluminescence imaging was used to monitor weekly tumor progression in mice across different treatment groups. **C** Survival analysis of experimental mice. **D** ELISA analysis of cytokine levels in peripheral blood plasma of mice from different groups. **E** Weight monitoring of experimental mice. **F** ALT and AST levels in experimental mice. **G** Histological analysis of vital organs in CAR-T treated mice. circRNA, circular RNA; *mRNA* messenger RNA, *CAR-T* chimeric antigen receptor T cell, *DLL3* Delta-like Ligand 3, *ELISA* enzyme linked immunosorbent assay, *ALT* alanine aminotransferase, *AST* aspartate transaminase

After successfully establishing the mouse lung orthotopic tumor model over 10 days, in both the experimental and control groups, 2 × 10^6 CAR-T cells and 2 × 10^6 normal T cells were administered via tail vein injection on Days 0, 4, 8, 12, 16, 20, 24, and 28, with bioluminescence imaging performed every 7 days (Fig. 6A). Bioluminescence imaging data indicated that CAR-T cells significantly delayed tumor progression compared to the control group. Among the CAR-T treatments, CAR-T cells engineered by circRNA showed more pronounced tumor growth inhibition compared to mRNA-engineered CAR-T cells, with two mice exhibiting complete tumor cell clearance (Figs. 6B 6C). Survival analysis revealed that all mice in the control group had died by Day 12, while in group of mRNA-engineered CAR-T cells, mice began dying between Days 24 and 33, with all eventually succumbing. In contrast, in the group of circRNA-engineered CAR-T cells, only one mouse died by Day 27, with the remaining mice surviving until the end of the experiment (Fig. 6D). ELISA data showed significantly higher levels of IL-2 and IFN-γ in the CAR-T groups compared to the control, with circRNA-engineered CAR-T cells yielding higher levels than mRNA-engineered CAR-T cells (Fig. 6E). Safety assessments indicated no significant weight changes in the experimental mice (Fig. 6F), with alanine aminotransferase (ALT) and aspartate transaminase (AST) levels slightly elevated but within normal ranges (Fig. 6G). Histological analysis of vital organs revealed no significant necrosis in CAR-T group (Fig. 6H). These results demonstrated that the anti-DLL3 CAR-T cells engineered by circRNA and electroporation show excellent anti-tumor efficacy in the SCLC orthotopic tumor model.

## Discussion

This study highlights the integration of circRNA technology with electroporation to construct CAR-T cells. Specifically, we successfully delivered anti-DLL3 CAR-encoding circRNA into primary human T cells using electroporation, resulting in the efficient generation of functional CAR-T cells. To provide a comparative framework, we also generated mRNA-based CAR-T cells. Remarkably, in vitro cytotoxicity assays revealed that circRNA-based CAR-T cells exhibited enhanced killing efficacy compared to the mRNA-based counterparts, particularly at low ET ratios. Furthermore, in both subcutaneous and orthotopic tumor models, circRNA-based CAR-T cells demonstrated robust antitumor activity. These findings establish the feasibility of using a circRNA-electroporation platform for CAR-T cell construction and present a novel therapeutic strategy targeting DLL3 for the treatment of SCLC.

Notably, mRNA-electroporation strategies have been explored extensively to generate CAR-T cells in the treatment of various diseases. A pilot phase I trial demonstrated that intravenous administration of mRNA-electroporated anti-cMET CAR T cells is both safe and feasible for metastatic melanoma and triple-negative breast cancer, with manageable toxicity and stable disease observed in some patients [32]. numerous preclinical studies have also confirmed the feasibility and safety of constructing CAR-T cells via mRNA-electroporation [33–36]. However, mRNA-based approaches face inherent challenges, including poor stability and high susceptibility to degradation, complicating transport and storage [37, 38]. Additionally, the relatively short duration of protein expression may necessitate repeated dosing to achieve the desired therapeutic efficacy [6, 7]. These limitations underscore the need for optimizing mRNA design or developing alternative RNA molecules with enhanced stability and translational efficiency to improve the effectiveness of RNA-based CAR-T therapies.

In this context, circRNA, with its enhanced stability and prolonged protein expression, has emerged as a promising next-generation alternative to mRNA [6, 7, 16, 39]. Currently, only a limited number of studies have investigated the feasibility and efficacy of using circRNA-electroporation strategy for CAR-T cell therapy. Recently, Hu et al. reported the development of anti-CD19 CAR-T cells using a circRNA-electroporation strategy [40]. Their in vitro and in vivo experiments in B-cell lymphoma demonstrated robust anti-tumor activity [40]. However, their focus on a well-established target in hematologic malignancies leaves the application of circRNA-electroporation-based CAR-T therapy in solid tumors unaddressed. To bridge this gap, our study focused on SCLC, employing the well-characterized target DLL3 and leveraging a circRNA-electroporation approach to construct anti-DLL3 CAR-T cells. Given the robust cytotoxicity and antitumor efficacy observed against SCLC, our findings suggested that CAR-T cells constructed through circRNA-electroporation represent a feasible and highly effective approach for the treatment of solid tumors.

In the realm of CAR-T therapy, an innovative strategy known as in vivo CAR-T therapy has recently gained attention. This approach involves the direct intravenous administration of LNPs encapsulating circRNA encoding the CAR structure into the peripheral blood [41]. The LNPs specifically target T cells, facilitating the intracellular delivery of circRNA and enabling the direct in situ generation of CAR-T cells within the body [41]. By bypassing the complex and labor-intensive ex vivo preparation process, this method holds the potential to significantly streamline CAR-T cell production [21]. Recent studies have demonstrated that mRNA-LNPs can successfully generate CAR-T cells in mice targeting the fibroblast activation protein, effectively reversing cardiac fibrosis and restoring heart function [42]. However, there is limited study reporting the in situ generation of CAR-T cells using circRNA-LNPs strategy. Building on our findings, which established the feasibility of circRNA-encoded CAR-T cells, we are currently advancing research into the in vivo CAR-T generation using circRNA-LNPs.

The use of electroporation-circRNA strategy to generate CAR-T cells represents a transformative approach in cancer immunotherapy, particularly for challenging malignancies such as SCLC [12]. To fully unlock the potential of this technology, several advancements are imperative. Refining circRNA design to enhance stability, improve translational efficiency, and minimize immunogenicity remains a critical priority [12, 43, 44]. Concurrently, the development of scalable and GMP-compliant manufacturing protocols is essential to facilitate clinical translation [45]. While electroporation offers robust transfection efficiency, alternative delivery platforms, such as LNPs, warrant exploration to further optimize therapeutic delivery and reduce cellular stress [46]. Future preclinical studies should focus on establishing long-term efficacy, biodistribution, and safety profiles to support clinical validation [47]. Moreover, the integration of circRNA-based CAR-T therapy with synergistic modalities, such as immune checkpoint inhibitors or tumor vaccines, holds promise for augmenting therapeutic efficacy and overcoming resistance mechanisms [48]. At last, expanding the range of tumor-specific targets beyond DLL3 could further broaden the scope of circRNA-based CAR-T applications to other malignancies. While challenges remain, this study highlights the substantial potential of circRNA-based CAR-T cells to redefine cancer immunotherapy. With sustained innovation and collaborative efforts, this platform could offer a versatile and powerful strategy for combating diverse cancers.

This study has certain limitations that warrant discussion. First, the absence of a viral-constructed CAR-T group as a comparator restricts the ability to comprehensively evaluate the relative therapeutic efficacy of circRNA-based CAR-T against SCLC models. Second, the study lacks the inclusion of more physiologically relevant models, such as organoids or patient-derived xenografts, which would better mimic the human tumor microenvironment and provide deeper insights into clinical translatability. Third, the technical challenges associated with establishing orthotopic tumor models resulted in a limited number of mice used in the study, potentially reducing the statistical robustness and generalizability of the findings. Nonetheless, this study represents a significant step forward in demonstrating the feasibility and therapeutic promise of circRNA-based CAR-T cells. Future efforts should focus on integrating viral-based control groups, utilizing advanced preclinical models that closely resemble human tumors, and refining experimental designs to overcome current technical constraints. These advancements will be crucial to further validate the clinical applicability and enhance the translational impact of this innovative approach.

In summary, this study successfully generated CAR-T cells by electroporating circRNA encoding anti-DLL3 CAR into human T cells. The engineered CAR-T cells demonstrated robust efficacy in targeting and eliminating SCLC cells. This research establishes the feasibility of the circRNA-electroporation-based CAR-T approach. Considering the limited effective treatment options and the poor prognosis associated with SCLC, there is a pressing need for more innovative and safer therapeutic strategies [49]. Our findings present a promising new avenue for the treatment of SCLC, offering potential improvements in therapeutic efficacy and patient outcomes.

## Supplementary Information

Additional file 1: Figure S1. High expression of DLL3 in SCLC tissues and cell lines. (A) Analysis using the GEO (GSE149507) database to assess DLL3 expression in SCLC tissues. (B) Investigation of DLL3 expression in SCLC cell lines using data from the CCLE database. (C) Immunohistochemical staining of SCLC and normal lung tissues for DLL3. (D-F) Validation of DLL3 expression in SCLC cell lines (H69, H524, and SHP-77) through WB, qPCR, and flow cytometry, respectively. (G) Immunofluorescence staining to determine DLL3 membrane localization in SCLC cells. DLL3, Delta-like Ligand 3; SCLC, small cell lung cancer; GEO, Gene Expression Omnibus; CCLE, Cancer Cell Line Encyclopedia; WB, western blot; qPCR, real time fluorescence quantitative polymerase chain reaction.

Additional file 2: Figure S2. Characterization and stability of circRNA encoding anti-DLL3 CAR. (A) Evaluation of protein expression from circRNA in 293T and H1299 cells using fluorescence, flow cytometry, and WB analysis. (B) Reverse-phase HPLC purification of circRNA products with chromatogram analysis. (C) Comparison of protein expression duration in H1299 cells transfected with circRNA and mRNA. (D) Immunogenicity assessment in 293T and H1299 cells transfected with circRNA and mRNA. (E) Stability test of circRNA and mRNA in different FBS gradients. (F) Intracellular stability assessment of circRNA and mRNA in H1299 cells over time. circRNA, circular RNA; mRNA, messenger RNA; CAR, chimeric antigen receptor; DLL3, Delta-like Ligand 3; HPLC, high performance liquid chromatography; FBS, fetus bovine serum; WB, western blot.

Additional file 3: Figure S3. Optimal electroporation voltage and dose for mRNA. (A) Electroporation voltage gradients (1400V, 1600V, and 1800V) were tested using fluorescence observation and flow cytometry to identify the optimal voltage for mRNA^EGFP^. (B) Flow cytometry was used to determine the optimal voltage for mRNA^Anti-DLL3 CAR^. (C) Dose gradients (1 µg, 2 µg, 3 µg, 4 µg, 5 µg, and 6 µg per million cells) were tested using fluorescence observation and flow cytometry to identify the optimal dose for mRNA^EGFP^. (D) Flow cytometry was used to determine the optimal dose for mRNA^Anti-DLL3 CAR^. mRNA, messenger RNA; CAR-T, chimeric antigen receptor T cell; DLL3, Delta-like Ligand 3; EGFP, enhanced green fluorescent protein; MFI, mean fluorescence intensity.

Additional file 4: Figure S4. Flow cytometry analysis of Tcm, Tem and Tscm subsets in CAR-T cells after coculture with SHP-77 cells. (A) Proportions of Tcm and Tem subsets. (B) Proportion of the Tscm subset. CAR-T, chimeric antigen receptor T cell; Tcm, central memory T cell; Tem, effector memory T cell; Tscm, stem cell-like memory T cell; mRNA, messenger RNA; circRNA, circular RNA.

Additional file 5: Figure S5. Cytotoxic efficacy experiments in GFP-H69 cell line. (A) Co-culture of DLL3-positive GFP-H69 cells with RNA-based anti-DLL3 CAR-T cells at various ET ratios, followed by fluorescence microscopy. (B) Flow cytometry analysis to assess cytotoxicity of circRNA^anti-DLL3 CAR-T^ and mRNA^anti-DLL3 CAR-T^ at different ET ratios. (C) qPCR analysis of cytokine expression in the co-culture system. (D) ELISA analysis of cytokine levels in co-culture supernatants. (E) Analysis of activated CD69+ and CD25+ T cells in the co-culture system. circRNA, circular RNA; mRNA, messenger RNA; GFP, green fluorescent protein; CAR-T, chimeric antigen receptor T cell; DLL3, Delta-like Ligand 3; ET, effector-to-target; qPCR, real time fluorescence quantitative polymerase chain reaction; ELISA, enzyme linked immunosorbent assay.

Additional file 6: Figure S6. Flow cytometry analysis of Tcm, Tem and Tscm subsets in CAR-T cells after coculture with H69 cells. (A) Proportions of Tcm and Tem subsets. (B) Proportion of the Tscm subset. CAR-T, chimeric antigen receptor T cell; Tcm, central memory T cell; Tem, effector memory T cell; Tscm, stem cell-like memory T cell; mRNA, messenger RNA; circRNA, circular RNA.

Additional file 7: Table S1. The primers, and sequences included in this study.

## Data Availability

No datasets were generated or analysed during the current study.
