# Supplementary figures and images for: Engineered circular RNA-based DLL3-targeted CAR-T therapy for small cell lung cancer
Source: Exp Hematol Oncol. 2025 Mar 12;14:35. doi: 10.1186/s40164-025-00625-8 (PMC11905684; doi:10.1186/s40164-025-00625-8)

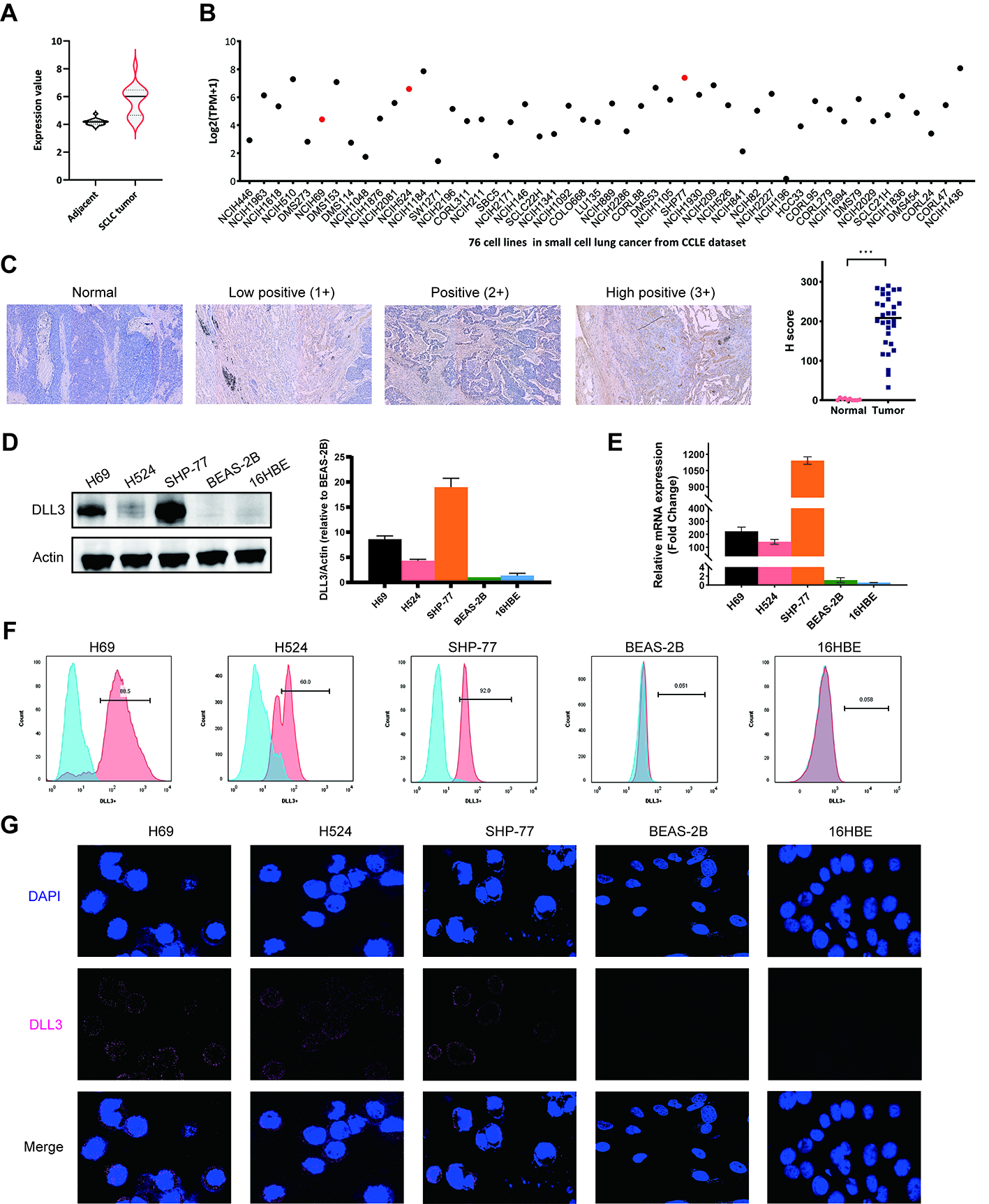

Supplement: Supplementary file 1 — Additional file 1: Figure S1. High expression of DLL3 in SCLC tissues and cell lines. (A) Analysis using the GEO (GSE149507) database to assess DLL3 expression in SCLC tissues. (B) Investigation of DLL3 expression in SCLC cell lines using data from the CCLE database. (C) Immunohistochemical staining of SCLC and normal lung tissues for DLL3. (D-F) Validation of DLL3 expression in SCLC cell lines (H69, H524, and SHP-77) through WB, qPCR, and flow cytometry, respectively. (G) Immunofluorescence staining to determine DLL3 membrane localization in SCLC cells. DLL3, Delta-like Ligand 3; SCLC, small cell lung cancer; GEO, Gene Expression Omnibus; CCLE, Cancer Cell Line Encyclopedia; WB, western blot; qPCR, real time fluorescence quantitative polymerase chain reaction. [file 40164_2025_625_MOESM1_ESM.tif]

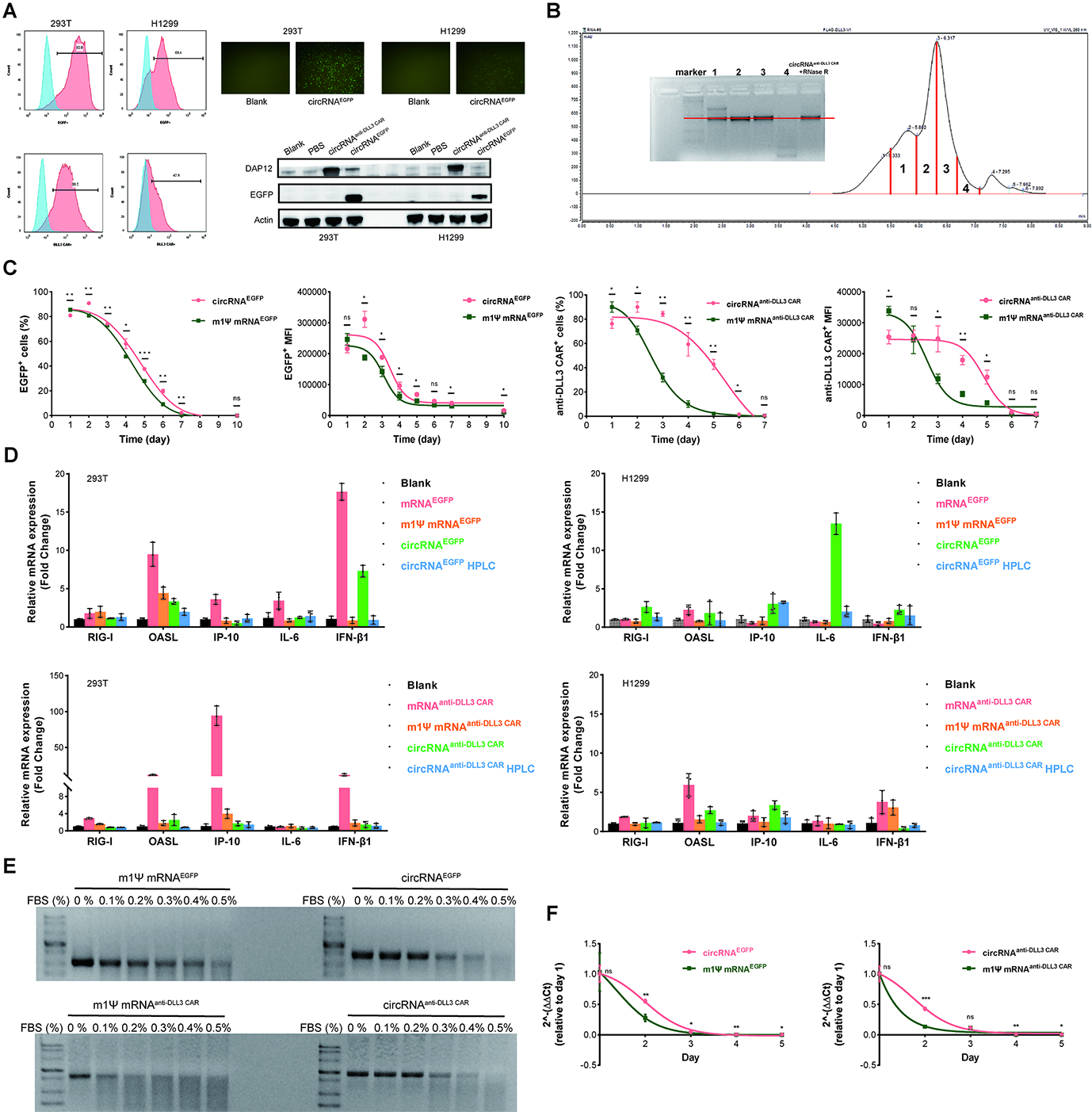

Supplement: Supplementary file 2 — Additional file 2: Figure S2. Characterization and stability of circRNA encoding anti-DLL3 CAR. (A) Evaluation of protein expression from circRNA in 293T and H1299 cells using fluorescence, flow cytometry, and WB analysis. (B) Reverse-phase HPLC purification of circRNA products with chromatogram analysis. (C) Comparison of protein expression duration in H1299 cells transfected with circRNA and mRNA. (D) Immunogenicity assessment in 293T and H1299 cells transfected with circRNA and mRNA. (E) Stability test of circRNA and mRNA in different FBS gradients. (F) Intracellular stability assessment of circRNA and mRNA in H1299 cells over time. circRNA, circular RNA; mRNA, messenger RNA; CAR, chimeric antigen receptor; DLL3, Delta-like Ligand 3; HPLC, high performance liquid chromatography; FBS, fetus bovine serum; WB, western blot. [file 40164_2025_625_MOESM2_ESM.tif]

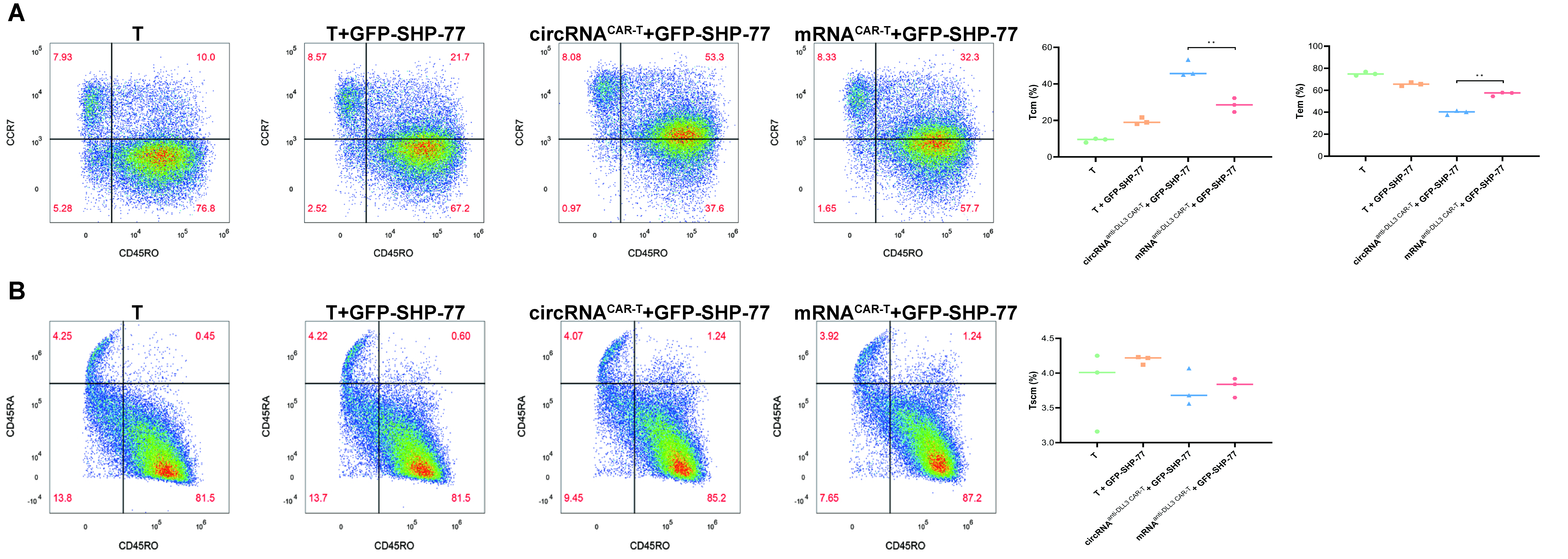

Supplement: Supplementary file 4 — Additional file 4: Figure S4. Flow cytometry analysis of Tcm, Tem and Tscm subsets in CAR-T cells after coculture with SHP-77 cells. (A) Proportions of Tcm and Tem subsets. (B) Proportion of the Tscm subset. CAR-T, chimeric antigen receptor T cell; Tcm, central memory T cell; Tem, effector memory T cell; Tscm, stem cell-like memory T cell; mRNA, messenger RNA; circRNA, circular RNA. [file 40164_2025_625_MOESM4_ESM.tif]
